# Cortical atrophy on baseline computed tomography imaging predicts clinical outcome in patients undergoing endovascular treatment for acute ischemic stroke

**DOI:** 10.1007/s00330-023-10107-2

**Published:** 2023-08-15

**Authors:** Gianluca Brugnara, Adrian Engel, Jessica Jesser, Peter Arthur Ringleb, Jan Purrucker, Markus A. Möhlenbruch, Martin Bendszus, Ulf Neuberger

**Affiliations:** 1 Department of Neuroradiology, Heidelberg University Hospital, Im Neuenheimer Feld 400, 69120 Heidelberg, Germany; 2 Division of Computational Neuroimaging, Heidelberg University Hospital, Heidelberg, Germany; 3 Department of Neurosurgery, Essen University Hospital, Essen, Germany; 4 Department of Neurology, Heidelberg University Hospital, Heidelberg, Germany

**Keywords:** Ischemic stroke, Thrombectomy, Prognosis

## Abstract

**Objective:**

Multiple variables beyond the extent of recanalization can impact the clinical outcome after acute ischemic stroke due to large vessel occlusions. Here, we assessed the influence of small vessel disease and cortical atrophy on clinical outcome using native cranial computed tomography (NCCT) in a large single-center cohort.

**Methods:**

A total of 1103 consecutive patients who underwent endovascular treatment (EVT) due to occlusion of the middle cerebral artery territory were included. NCCT data were visually assessed for established markers of age-related white matter changes (ARWMC) and brain atrophy. All images were evaluated separately by two readers to assess the inter-observer variability. Regression and machine learning models were built to determine the predictive relevance of ARWMC and atrophy in the presence of important baseline clinical and imaging metrics.

**Results:**

Patients with favorable outcome presented lower values for all measured metrics of pre-existing brain deterioration (*p* < 0.001). Both ARWMC (*p* < 0.05) and cortical atrophy (*p* < 0.001) were independent predictors of clinical outcome at 90 days when controlled for confounders in both regression analyses and led to a minor improvement of prediction accuracy in machine learning models (*p* < 0.001), with atrophy among the top-5 predictors.

**Conclusion:**

NCCT-based cortical atrophy and ARWMC scores on NCCT were strong and independent predictors of clinical outcome after EVT.

**Clinical relevance statement:**

Visual assessment of cortical atrophy and age-related white matter changes on CT could improve the prediction of clinical outcome after thrombectomy in machine learning models which may be integrated into existing clinical routines and facilitate patient selection.

**Key Points:**

*• Cortical atrophy and age-related white matter changes were quantified using CT-based visual scores.*

*• Atrophy and age-related white matter change scores independently predicted clinical outcome after mechanical thrombectomy and improved machine learning–based prediction models.*

*• Both scores could easily be integrated into existing clinical routines and prediction models.*

**Supplementary information:**

The online version contains supplementary material available at 10.1007/s00330-023-10107-2.

## Introduction

Endovascular therapy (EVT) is the standard treatment for patients with acute ischemic stroke (AIS) due to large vessel occlusion up to 24 h after onset [1, 2], with recent evidence suggesting a potential treatment efficacy even for patients with low Alberta Stroke Program Early CT score (ASPECTS) at admission [3–5].

Several factors are known to influence the potential recovery of patients after treatment, and a successful and timely recanalization is often not self-sufficient to explain the final functional status, despite having a large influence on the clinical outcome [6]. Besides various clinical and treatment parameters, the pre-morbid status of patients is known to be particularly influential on the final clinical outcome and must always be considered when building predictive models [7–9]. In practice, the pre-stroke status is generally evaluated by recording the patient’s functional independence (i.e., through the modified Rankin Scale—mRS), age, and the presence or absence of other pre-stroke comorbidities (for example, diabetes or hypertension).

Alongside all these factors, previous studies have also shown the importance of chronic brain deterioration and reduced brain reserve on the final patient’s recovery after AIS, and demonstrated that the presence of chronic vascular insults and small vessel disease (SVD) [10–12] is associated with worse outcome and long term recovery [12]. Cortical atrophy has also been demonstrated to negatively influence clinical outcome after EVT [13–17] as well as to increase the likelihood of post-stroke cognitive decline [18, 19]. These factors are particularly relevant considering the increasing evidence suggesting and/or supporting the use of EVT over medical management also in elderly patients [20, 21].

In this context, brain atrophy has been mostly assessed using MRI data and automated measurements, both of which may not be readily available or cost-effective, especially in smaller centers. Moreover, while previous studies were able to prove the value of brain atrophy as an independent predictor, they did not investigate its overall impact on outcome prediction models, especially when combined alongside all other available information through machine learning algorithms.

Here, we independently validated the impact of pre-existing SVD and atrophy on clinical outcome after EVT for AIS in a large, retrospective single-center dataset. We aimed to assess these factors through simple CT-based visual rating scales of age-related white matter changes (ARWMC) and cortical atrophy, which could be broadly applicable in clinical practice, and to investigate their overall relevance for machine learning-based predictive modeling when combined with all available information at baseline. Furthermore, we investigated their impact on post-discharge improvements in functional status.

## Methods

The study was approved by the local ethics committee, and the requirement for informed consent was waived (S-784/2018).* n* = 1103 consecutive patients with confirmed AIS from large vessel occlusion (LVO) affecting the middle cerebral artery (MCA) territory who underwent imaging and EVT at the Department of Neuroradiology of the Heidelberg University Hospital between 01/2013 and 11/2019 were retrospectively enrolled for analysis.

### Imaging, endovascular treatment, and clinical assessment

Patients underwent imaging with a 64-slice SOMATOM Definition AS CT scanner (Siemens Healthcare GmbH) as described previously [7]. The complete diagnostic and interventional methods are included in the Data Supplement. Briefly, native cranial computed tomography (NCCT) was acquired with standard initial parameters of 120 kV and 20 mAs, which were then automatically adapted slice-wise using the CARE Dose 4D automatic exposure control system (Siemens Healthineers) and iteratively reconstructed with a J40s kernel (low pass filter—smooth, soft tissue kernel) at 1 mm (*n* = 485, 43%) and 4 mm (*n* = 1103 patients, 100%) slice thickness. NCCT was acquired at admission and the decision for endovascular treatment as well as the administration and dosing of recombinant human-tissue plasminogen activator (rtPA) was individually made for each patient based on a consensus between the treating neurologist and neurointerventionalist, following national and international guidelines [1, 2]. The following EVT was performed with a biplane angiographic system (Artis Zee Biplane and Artis Q, Siemens Healthineers). Routine follow-up NCCT was performed on the same scanner within 18 to 36 h or earlier in case of clinical deterioration for all patients.

### Radiological assessment of pre-existing brain deterioration

**Fig. 1:**
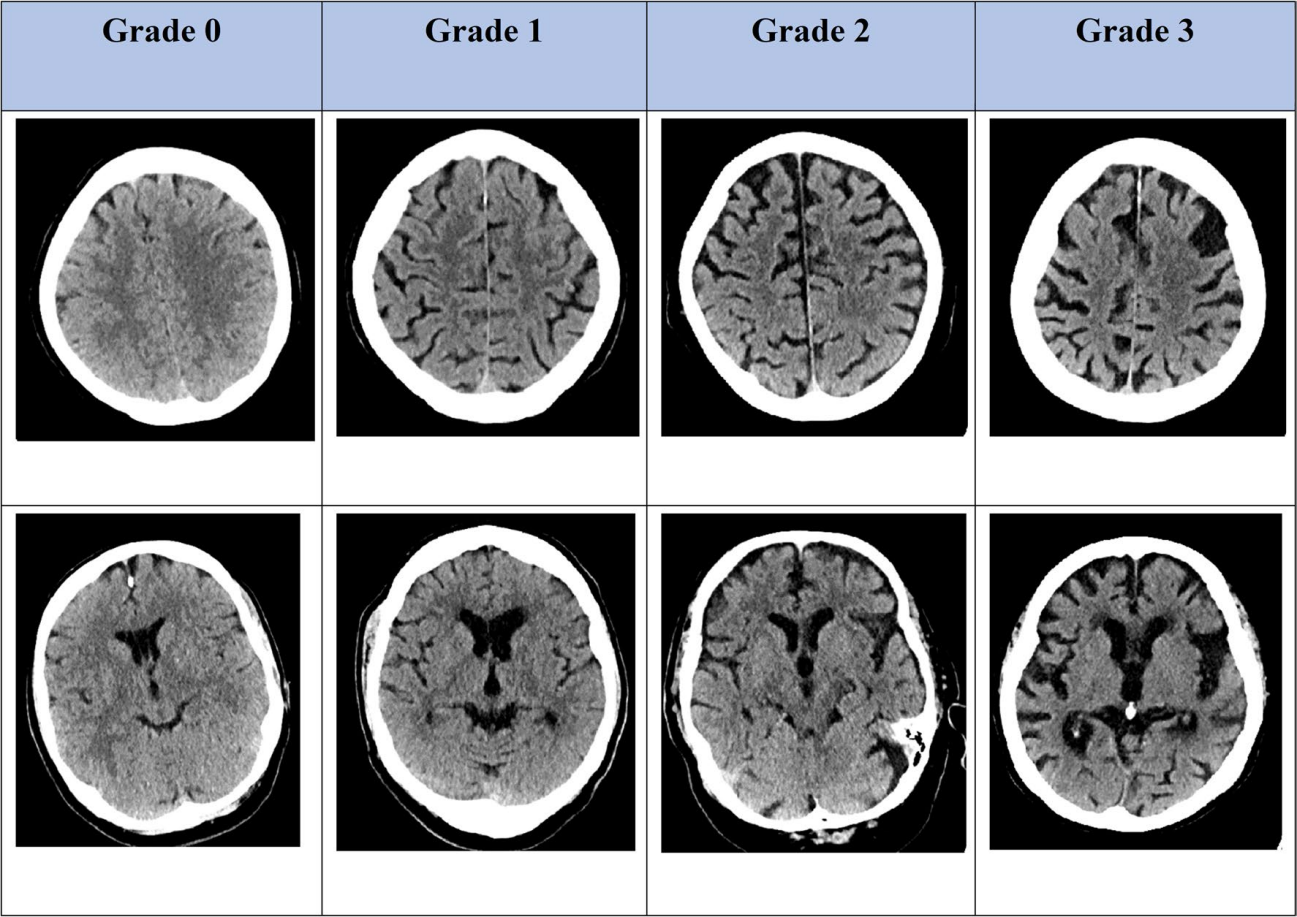
Visual examples for atrophy grading on CT images. Grade 1—minor atrophy to the insular and/or the frontoparietal region, cerebral sulci slightly more pronounced. Grade 2—moderate atrophy, further widening of the cerebral sulci. Grade 3—global atrophy, severely widened sulci, thinning of the cortical gyri. Windowing was set at L40/W60 for all images

Baseline NCCT was visually reviewed by AE (radiology resident, 3 years of experience) and UN (board-certified neuroradiologist, 8 years of experience) to determine (i) age-related white matter changes (ARWMC) of periventricular white matter and basal ganglia [22], (ii) presence/absence of previous strokes, (iii) Evans’ index[23], and (iv) maximal diameter of the third ventricle. Additionally, cortical atrophy was assessed using a four-point scale by both readers to subsequently assess the inter-observer variability of our method, as depicted in Fig. 1, also based on a previous publication on the topic [24]. Briefly, pathological grades were defined as follows: Grade 1—minor atrophy to the insular and/or the frontoparietal region, cerebral sulci slightly more pronounced. Grade 2— moderate atrophy, further widening of the cerebral sulci. Grade 3—global atrophy, severely widened sulci, thinning of the cortical gyri.

### Further clinical, imaging, and angiographic parameters

Clinical data were collected by a certified stroke neurologist. Baseline epidemiological and clinical characteristics were included in the study (sex, age, pre-morbid mRS score, NIHSS score on admission, concomitant treatment with intravenous rtPA, comorbidities [hypertension, dyslipidemia, coronary heart disease, atrial fibrillation, diabetes mellitus including serum blood levels for HbA1c and glucose]) as well as interventional angiographic characteristics (interval from symptom onset to groin puncture and from groin puncture to recanalization, extent of recanalization as assessed by the treating neurointerventionalist with the modified treatment in cerebral ischemia (mTICI score) and post-interventional clinical characteristics (NIHSS score after 24 h). The Alberta Stroke Program Early CT Score (ASPECTS) was evaluated automatically through e-ASPECTS (Brainomix) on 1 mm slices and visually reviewed by AE (radiology resident with 2 years of experience) and UN (board-certified neuroradiologist with 8 years of clinical experience) [25]. Follow-up NCCTs were visually assessed for follow-up ASPECTS as well as the presence of intracranial hemorrhages (ICH) (scored according to the Heidelberg Bleeding Classification—HBC) [26]. The correlation of neurological deterioration with imaging findings was assessed by reviewing clinical data (e.g., medical records) to further classify ICH as symptomatic ICH or asymptomatic ICH, in compliance with operational guidelines of HBC.

The functional outcome was assessed with the mRS score at 90 days (mRS90) and was classified as “favorable” outcome for patients with a mRS90 ≤ 2 or an mRS at 90 days equal to the pre-stroke mRS. A NIHSS score of 42 at discharge was assigned to patients who deceased during their initial hospitalization.

### Statistical analysis

Statistical analyses were performed in R version 4.0.3. Metrics were tested for normality using the Shapiro–Wilk test, and Wilcoxon, t-test, or chi-squared test were then used accordingly depending on the nature of the metrics. The intraclass correlation coefficient (ICC) was calculated for the visual assessment of cortical atrophy between the two expert raters. Spearman’s rho was used to assess the correlation between variables.

Logistic regression as well as ordinal logistic regression were performed to assess the effects of the various measures of pre-existing brain deterioration on outcome while adjusting for other covariates, as listed below. The metrics were further tested in machine learning models. Briefly, these were built using the caret package [27] and based on gradient-boosting machine classifiers with the 0.632 bootstrapping procedure for cross-validation [28], as further described in the Data Supplement. Synthetic minority over-sampling (SMOTE) was applied to correct for sample imbalance. Model performance was assessed through ROC curves, and significant differences between the performance of the developed models were then assessed using DeLong’s test. The variable importance of parameters within the machine learning models was then assessed through random forest variable importance.

Within predictive modelling performed with either logistic regression, ordinal regression, and gradient boosting classifiers, three sets of metrics were present in the dataset in addition to ARWMC and cortical atrophy.

1. **Baseline metrics: **age, sex, stroke laterality (right vs. left), diabetes, coronary heart disease (CHD), hypertension, atrial fibrillation, dyslipidemia, i.v. rtPA lysis administration, baseline NIHSS, pre-morbid mRS, baseline ASPECTS, baseline glucose, baseline HbA1c, time from stroke onset to first imaging exam
2. **Post-interventional metrics**: successful recanalization (yes/no), complete recanalization (TICI ≥ 2b [yes/no]), NIHSS at 24 h post-intervention, presence of bleeding at follow-up imaging, time from groin puncture to final TICI score, number of thrombectomy maneuvers, follow-up ASPECTS
3. **Post-discharge metrics**: NIHSS at discharge, mRS at discharge

## Results

Across the study cohort, *n* = 414 patients (38%) presented a favorable clinical outcome, and *n* = 689 patients with an unfavorable outcome at 90 days (mRS ≤ 2 or unchanged as compared to pre-morbid). For our secondary analysis, *n* = 284 patients (26%) presented an improvement in mRS at 90 days as compared to the mRS collected at hospital discharge. The different clinical and epidemiological characteristics between patients presenting favorable vs. unfavorable clinical outcome at 90 days are listed in Table 1. Briefly, the two groups presented significant differences in all listed metrics (*p* < 0.01) aside from the presence/absence of hypertension (*p* = 1.00), dyslipidemia (*p* = 0.894), and the distribution of patients’ sex (*p* = 0.079).

**Table 1:** Epidemiological and clinical statistics for patients with favorable (mRS ≤ 2) vs. unfavorable (mRS > 2) clinical outcome at 90 days. Metrics are listed as median (IQR) for continuous variables, and number (%) for ordinal variables. In univariate tests, the two cohorts presented significant differences in all listed metrics aside from the presence/absence of hypertension and dyslipidemia

Parameter	Good outcome (*n* = 414)	Poor outcome (*n* = 689)	*p* value
Age, years	73 (67–80)	79 (73–84)	***p* < 0.001**
Sex, female	219 (53%)	404 (58%)	*p* = *0.079*
Hypertension	320 (77%)	533 (77%)	*p* = 1.00
Diabetes mellitus	69 (17%)	175 (25%)	***p* < 0.001**
Atrial fibrillation	172 (42%)	352 (51%)	***p* < 0.01**
Dyslipidemia	128 (31%)	217 (31%)	*p* = 0.894
Coronary heart disease	91 (22%)	192 (28%)	***p* = 0.036**
Pre-morbid mRS	0 (0–2)	1 (0–3)	***p* < 0.001**
NIHSS score	13 (7–18)	18 (13–22)	***p* < 0.001**
Serum glucose, mg/dL	113 (99–133)	124 (105–146)	***p* < 0.001**
HbA1c	5.7 (5.4–6.1)	5.8 (5.5–6.3)	***p* < 0.001**
Baseline ASPECTS	9 (8–10)	8 (7–10)	***p* < 0.001**
i.v. rtPA	235 (57%)	330 (48%)	***p* < 0.01**
Onset-to-recanalization, min	294 (206–451)	370 (269–578)	***p* < 0.001**
Puncture-to-recanalization, min	48 (32–77)	76 (47–119)	***p* < 0.001**
Thrombectomy maneuvers	1 (1–2)	2 (1–3)	***p* < 0.001**
Complete recanalization (mTICI ≥ 2c)	259 (63%)	283 (41%)	***p* < 0.001**
NIHSS after 24 h	4 (2–7)	18 (11–24)	***p* < 0.001**
Shift of NIHSS after 24 h	− 8 (− 12 to − 3)	0 (–5 to + 6)	***p* < 0.001**
NIHSS at discharge	2 (0–4)	16 (9–23)	***p* < 0.001**
mRS at discharge	2 (1–3)	4 (4–5)	***p* < 0.001**
mRS after 90 days	2 (1–2)	5 (4–6)	***p* < 0.001**
Mortality after 90 days	0 (0%)	243 (32%)	***p* < 0.001**

Significant values are highlighted in bold

An overview of the distribution of all measured metrics of pre-existing brain deterioration is provided in Table 2. Here, patients with an unfavorable clinical outcome at 90 days presented a higher rate of previous strokes (*p* = 0.010) as well as significant differences in all measures of pre-existing brain deterioration, with higher basal ganglia and periventricular ARWMC (*p* = 0.014 and *p* < 0.001, respectively), higher cortical atrophy scores (*p* < 0.001), Evans’ Index (*p* < 0.001), and third ventricle diameter (*p* < 0.001).

**Table 2:** Measures of pre-existing brain deterioration for patients with favorable (mRS ≤ 2) vs. unfavorable (mRS > 2) clinical outcome at 90 days. Metrics are listed as median (IQR) for continuous variables, and number (%) for ordinal variables. Wilcoxon Rank-sum test was used for continuous variables, and the chi-squared test was used for categorical variables

Parameter	Good outcome (*n* = 414)	Poor outcome (*n* = 689)	*p* value
ARWMC (basal ganglia)			
Score = 0	171 (41%)	259 (38%)	***p* = *0.014***
Score = 1	159 (38%)	244 (35%)	
Score = 2	70 (17%)	132 (19%)	
Score = 3	14 (3%)	54 (8%)	
ARWMC (periventricular)			
Score = 0	103 (25%)	68 (10%)	***p* < *0.001***
Score = 1	162 (39%)	251 (36%)	
Score = 2	102 (25%)	190 (28%)	
Score = 3	47 (11%)	180 (26%)	
Cortical atrophy (CT scale)			
Score = 0	79 (19%)	61 (9%)	***p* < *0.001***
Score = 1	178 (43%)	208 (30%)	
Score = 2	133 (32%)	251 (36%)	
Score = 3	24 (6%)	169 (25%)	
Evans’ Index	0.29 (0.26–0.31)	0.29 (0.27–0.32)	***p* < *0.001***
Third ventricle diameter (mm)	9 (7–11)	10 (8–12)	***p* < *0.001***
Previous stroke			
Yes	21 (5%)	66 (10%)	***p* = *0.010***
No	393 (95%)	623 (90%)	

Significant values are highlighted in bold

The related inter-rater analysis for the cortical atrophy measurements revealed a moderately high ICC of 0.706 (95% CI 0.673–0.736), suggesting a good reproducibility. Correlation analyses for both ARWMC and cortical atrophy revealed only weak to average correlations with the patient’s age, with rho = 0.385 (*p* < 0.001) for periventricular ARWMC, rho = 0.285 (*p* < 0.001) for basal ganglia ARWMC, and rho = 0.434 (*p* < 0.001) for cortical atrophy. Cortical atrophy presented a weak to average correlation with mRS at 90 days (rho = 0.360, *p* < 0.001).

Patients with hypertension (*p* < 0.001), coronary heart disease (*p* < 0.05), and atrial fibrillation (*p* < 0.001) presented significantly higher score distributions for both ARWMC and cortical atrophy, as further listed in Table 3, whereas no significant differences were noted for the presence of diabetes and dyslipidemia (*p* > 0.05).

**Table 3:** Multivariable logistic regression model for prediction of favorable vs. unfavorable outcome at 90 days after EVT. ARWMC, cortical atrophy, hypertension, baseline NIHSS, pre-morbid mRS, baseline ASPECTS, and time from onset to first imaging exam were all shown to be independent predictors for clinical outcome, as listed above. LR = Likelihood Ratio Test

Logistic regression model (good vs. poor outcome at 90 days)	adj. OR (95%CI)	*p* (Wald's test)	*p* (LR test)
ARWMC (basal ganglia)			
Grade 1	0.95 (0.64–1.41)	0.810	0.968
Grade 2	1.06 (0.62–1.80)	0.832	
Grade 3	1.05 (0.46–2.38)	0.911	
ARWMC (periventricular)			
Grade 1	0.47 (0.29–0.76)	**0.002**	**0.006**
Grade 2	0.64 (0.36–1.13)	**0.121**	
Grade 3	0.43 (0.22–0.85)	**0.015**	
Cortical atrophy scale			
Grade 1	0.90 (0.55–1.48)	0.684	** < 0.001**
Grade 2	0.67 (0.39–1.16)	0.150	
Grade 3	0.27 (0.13–0.54)	** < 0.001**	
Age	0.99 (0.98–1.00)	0.141	0.143
Sex	0.95 (0.70–1.28)	0.728	0.728
Laterality (right vs. left)	0.94 (0.70–1.27)	0.704	0.704
Diabetes	0.98 (0.61–1.56)	0.926	0.926
CHD	0.82 (0.58–1.17)	0.273	0.272
Hypertension	1.54 (1.06–2.22)	**0.022**	**0.021**
Atrial fibrillation	1.03 (0.76–1.40)	0.847	0.847
Dyslipidemia	1.01 (0.72–1.39)	0.998	0.998
i.v. rtPA	1.10 (0.81–1.50)	0.528	0.528
Baseline NIHSS	0.91 (0.89–0.94)	** < 0.001**	** < 0.001**
Pre-morbid mRS	0.85 (0.74–0.97)	**0.017**	**0.016**
Baseline ASPECTS	1.34 (1.20–1.49)	** < 0.001**	** < 0.001**
Glucose	0.99 (0.99–0.99)	**0.018**	**0.018**
HbA1c	0.81 (0.66 –0.99)	**0.035**	**0.027**
Time from onset to the first imaging	0.99 (0.99–0.99)	**0.003**	**0.002**

Significant values are highlighted in bold

### Regression analysis

After performing an exploratory multivariable logistic regression with the inclusion of all brain deterioration metrics (but no confounders), ARWMCs and cortical atrophy emerged as the most relevant predictors (*p* < 0.001) and were included in subsequent predictive models, while other metrics were discarded.

Multivariable logistic regression with the inclusion of all clinical and imaging baseline parameters revealed the independent predictive significance of both cortical atrophy (adj. OR for grade 3–0.27 [0.13–0.54], *p* < 0.001) and periventricular ARWMC (adj. OR for grade 3–0.43 [0.22–0.85], *p* < 0.05) for predicting a favorable clinical outcome at 90 days, with adjusted OR for each class as well as the other independent predictors of outcome further listed in Table 3.

**Fig. 2:**
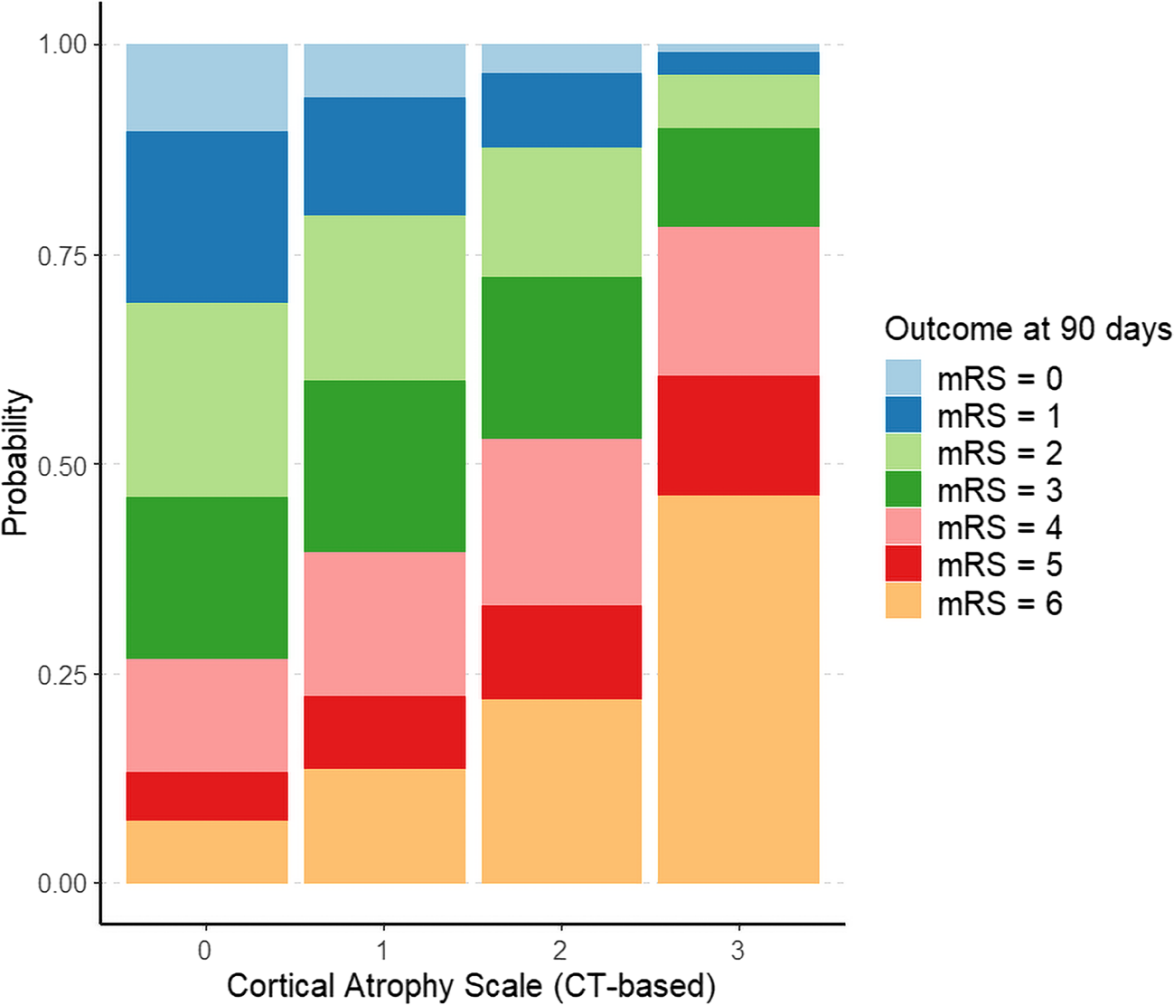
Cumulative probability for each mRS score class at 90 days (mRS 0–6) sorted by cortical atrophy score as calculated through ordinal logistic regression, demonstrating a higher likelihood for a worse clinical outcome for patients with higher atrophy scores, independently of all other included variables

Ordinal logistic regression was then performed with the goal of predicting the ordinal, non-dichotomous mRS score at 90 days, and demonstrated again the relevance of cortical atrophy to independently predict outcome as measured by increases in mRS (e.g. adj. OR for grade 3 atrophy—3.13 [1.89–5.37], *p* < 0.001), while ARWMC did not reach significance (*p* > 0.05), as further listed in Supplemental Table S1. Figure 2 depicts the cumulative probabilities for each mRS class based on the patient’s atrophy scale, showing a linear increase of likelihood for higher mRS scores and death (mRS = 6) parallel to the stepwise increases in the atrophy scale.

### Machine learning models

The addition of both cortical atrophy and periventricular ARWMC to the other available baseline clinical and imaging metrics led to a minor but significant improvement in the prediction performance when using machine learning models on the cross-validation sample (*p* < 0.001), with an AUC of 0.775 (95% CI, 0.772–0.779), as compared to 0.763 (0.760–0.766) without ARWMC and atrophy scale—see Supplemental Figure S1 for a depiction of the ROC curves. Both ARWMC and atrophy were among the top-10 predictors used by the model after inclusion, as depicted in Supplemental Figure S2.

We then developed three multivariable logistic regression models for predicting mRS improvement after hospital discharge, with (i) baseline, (ii) baseline + post-EVT, and (iii) baseline + post-EVT + post-discharge metrics. As listed in Supplemental Table S2, cortical atrophy maintained its relevance as an independent predictor of clinical improvement post-discharge across all tested models (*p* < 0.001) despite the addition of post-interventional and even post-discharge metrics.

## Discussion

Multiple variables beyond the extent of recanalization can impact the clinical outcome after AIS due to LVO. Here, we could confirm that both ARWMC and cortical atrophy were independent predictors of clinical outcome at 90 days also when controlled for all other baseline confounders, and the probability of a worse outcome or death increased linearly with the severity of atrophy. ARWMC and atrophy were only moderately correlated with patient’s age but presented significantly higher scores in patients with cardiovascular risk factors (namely hypertension, CHD, and atrial fibrillation). Scoring of cortical atrophy was performed using a simple and novel 4-point NCCT-based scale which demonstrated a good inter-observer agreement between our two raters, suggesting good reproducibility of our method. Overall, the inclusion of cortical atrophy and ARWMC into machine learning models for outcome prediction led to a minor but significant performance improvement in prediction accuracy, and both ARWMC and atrophy were considered among the top-10 predictors by the algorithm. Finally, atrophy had a predictive effect on a potential improvement of mRS after hospital discharge, even in the presence of all other post-interventional metrics.

Our results are consistent with other findings in the literature which previously demonstrated the independent predictive significance of atrophy [13–17] as well as ARWMC and SVD [9–12, 29] onto the final clinical outcome of patients undergoing EVT, and support a relevant underlying contribution of pre-stroke brain deterioration beyond all other examined parameters. This further corroborates the longstanding concept that patients with lower brain reserve would present an implicit disadvantage in compensating for the damage sustained through AIS [9, 30].

Moreover, the predictive effect of atrophy for clinical improvements after hospital discharge hints at the fact that the true role of cerebral atrophy might be played out on a longer time scale and/or in the rehabilitation phase, also considering the well-known post-stroke cognitive decline which inevitably affects some patients at later stages [18, 19].

As compared to previous works in the literature which relied on MRI-based and/or automated volumetric methods to assess atrophy [13, 14], we decided to use a simple 4-point grading scale performed visually on NCCT to grade cortical atrophy. While our method cannot implicitly be as reproducible or accurate as automated volumetric methods performed on MRI, it compensates for this issue through its simplicity, which makes it broadly applicable in clinical practice with minimal training, ultimately increasing the amount of information available to the treating physician even in smaller institutions where MRI or research IT infrastructures with specialized image analysis tools might not be available or cost-effective. Moreover, our data demonstrated a good inter-rater agreement in the atrophy scores, suggesting that the method could be implemented by other institutions.

Nonetheless, our data also showed that the addition of ARWMC and atrophy only led to a minor improvement in prediction performance with machine learning-based models that encompassed all available baseline data, despite their independent predictive effect. This aspect was never tested in the available literature, to the best of our knowledge. Overall, large leaps in performance are not to be expected with the addition of new parameters in the context of multimodal predictive modeling of AIS due to the complex multifactorial interactions that determine the final outcome, and existing models struggle to increase baseline performance due to the large number of confounders which are present throughout the subsequent treatment, hospital stay and rehabilitation [7, 8]. Taking these factors into consideration, atrophy and ARWMC achieved a significant result after the inclusion in our model, as they allowed to push the performance even further than what could be achieved with the previous inclusion of cardiovascular risk factors and other indicators of pre-morbid patient status, to which they are inevitably related. Due to the simplicity of our NCCT-based scoring system and the implicit availability of imaging for AIS patients, measures of ARWMC and atrophy could then be easily integrated into the evaluation of the patient status at baseline on a broad scale.

Our study presents some limitations. First, although we included a large patient sample, we used a retrospective, single-center study design. Further investigations and validations with a multi-centric dataset or prospective data might confirm our results and provide more definite evidence. Second, while we tried to assess the reproducibility of our NCCT atrophy score, we only had two raters at our disposal; further large-scale testing with a higher number of raters is warranted in order to precisely assess the variability of our method. Third, we did not use a test set or perform external validation with our machine learning models; although this is to be considered a major drawback in machine learning studies, our aim was not that of producing a high-performing and reproducible tool that could be applied in other centers, but rather to perform an indirect investigation on the potential added benefits of ARWMC and atrophy. Testing samples were therefore not implicitly required for the purpose of this study. Fourth, we did not validate our results against MRI volumetry as we lack access to a patient cohort that received both examinations. Further targeted studies with this type of design might aid in enhancing our NCCT-based score and its reproducibility. Lastly, our models did not include data derived from CT perfusion imaging, as this is performed only in selected cases at our institution; moreover, previous works have demonstrated the limited utility of CT perfusion for outcome prediction in the presence of all other available baseline information [7] and the correlation between ischemic core and ASPECTS [31–33].

In conclusion, cortical atrophy and ARWMC were strong independent predictors of clinical outcome after EVT in our retrospective dataset, and their inclusion in a prediction model using established parameters led to a significant improvement in prediction accuracy. Moreover, cortical atrophy could independently predict an improvement in mRS after hospital discharge. Both scores could easily be integrated into existing clinical routines and prediction models given the simple NCCT-based visual rating method in order to improve the selection of eligible patients and resource allocation.

### Supplementary Information

Below is the link to the electronic supplementary material.

Supplementary file1 (PDF 361 KB)

## Abbreviations

AIS: Acute ischemic stroke
ARWMC: Age-related white matter changes
ASPECTS: Alberta Stroke Program Early CT score
AUC: Area under the curve
CHD: Coronary heart disease
CTA: Computed tomography angiography
EVT: Endovascular therapy
HBC: Heidelberg Bleeding Classification
ICC: Interrater correlation coefficient
ICH: Intracranial hemorrhage
IQR: Interquartile range
LVO: Large vessel occlusion
MCA: Middle cerebral artery
mRS: Modified Rankin Scale
mTICI: Modified Thrombolysis in Cerebral Infarction score
NCCT: Native cranial computed tomography
NIHSS: National Institute of Health Stroke Scale
OR: Odds ratio
ROC: Receiver operating characteristics
rtPA: Recombinant human-tissue plasminogen activator
SMOTE: Synthetic minority over-sampling
SVD: Small vessel disease

## Declarations

### Guarantor

The scientific guarantor of this publication is Ulf Neuberger.

### Statistics and biometry

No complex statistical methods were necessary for this paper.

### Study subjects or cohorts overlap

Some study subjects or cohorts have been previously reported in Brugnara G, Mihalicz P, Herweh C, et al (2022), https://doi.org/10.1136/jnis-2022-019400.

### Methodology

• Retrospective

• diagnostic or prognostic study

• performed at one institution
