# Supplementary material for: Cortical atrophy on baseline computed tomography imaging predicts clinical outcome in patients undergoing endovascular treatment for acute ischemic stroke
Source: Eur Radiol. 2023 Aug 15;34(2):1358–66. doi: 10.1007/s00330-023-10107-2 (PMC10853300; doi:10.1007/s00330-023-10107-2)
Supplement: Supplementary file 1 — Supplementary file1 (PDF 361 KB) [file 330_2023_10107_MOESM1_ESM.pdf]

Cortical atrophy on baseline computed tomography imaging predicts clinical outcome in patients undergoing endovascular treatment for acute ischemic stroke

Electronic Supplementary Material

Diagnostic and Intervention:

Native CT and CT-angiography (CTA) were acquired with 120 kV and 20 mAs (which were then automatically adapted slice-wise using the CARE Dose 4D automatic exposure control system (Siemens Healthineers)), and for CTA a single contrast bolus of 65ml Xenetix 350 (Guerbet) was given at a flow rate of 4.0 ml/s, followed by a 20 ml saline chaser. CCT sequences were reconstructed with a J40s kernel at 1 mm and 4mm slice thickness and CTA was reconstructed with both the B26f and B46f kernels at a slice thickness of 0.75mm.

Cranial CT (CCT) was acquired at admission and the decision for endovascular treatment as well as the administration and dosing of rtPA was individually made for each patient based on a consensus between the treating neurologist and neurointerventionalist, following national and international guidelines[1, 2]. The following EVT was performed with a biplane angiographic system (Artis Zee Biplane and Artis Q, Siemens Healthineers). Transfemoral arterial access was used in all cases, and as a standardized approach, a guide catheter was placed within the proximal internal carotid artery, where an intermediate catheter was then introduced. The choice of materials for the procedure (e.g., (balloon-) guide catheters, intermediate and (micro-) catheters, (micro-) guidewires, and stent-retriever) was at the discretion of the treating neurointerventionalist based on the availability of materials, and subject to change according to the technical developments and standard of knowledge over the data collection timeframe; the same applied to the techniques used for mechanical thrombectomy, including the choice to perform mechanical thrombectomy either using a combined approach with stent-retriever thrombectomy and distal aspiration or direct thromboaspiration as a first-line approach. Routine follow-up CCT was performed on the same scanner within 18 to 36 hours or earlier in case of clinical deterioration for all patients.

Machine learning models:

Gradient boosting algorithms are an extremely popular machine learning algorithm that have proven successful across many domains [1; 2]. These algorithms produce a prediction model in the form of an ensemble of weak prediction models, typically decision trees. Gradient boosting thereby starts with a weak model (a decision tree with only a few splits) and sequentially boosts its performance by continuing to build new trees, where each new tree in the sequence tries to fix up where the previous one made the biggest mistakes (i.e., each new tree in the sequence will focus on the training rows where the previous tree had the largest prediction errors). We used the caret library [3] in R for generating the gradient boosting classifiers. Model development, including the selection of predictors, hyperparameter tuning and validation was performed with a 0.632 bootstrap approach which provides stable and nearly unbiased estimates of performance [4; 5]. Bootstrapping is a resampling method that allows one to make inferences about the population that the sample originated from by drawing B samples (B = 25 in the current study) with replacement from the original dataset, of the same size as the original dataset. In the .632 bootstrap method, a model is built for each bootstrap sample and evaluated only in those subjects not sampled. The prediction errors are then averaged over all bootstrap samples (test performance). As the dataset is sampled with replacement, on average 63.2% of the subjects are included at least once in a bootstrap sample, giving the method its name. The estimated performance is a weighted combination of the apparent performance (resubstitution error estimate on the full dataset) and test performance.

1 Natekin A, Knoll A (2013) Gradient boosting machines, a tutorial. Front Neurorobot 7:21

2 Lu H, Praneeth Karimireddy S, Ponomareva N, Mirrokni V (2019) Accelerating Gradient Boosting Machine.

3 Kuhn M (2008) Building Predictive Models inRUsing thecaretPackage. Journal of Statistical Software 28:26

4 Steyerberg EW (2009) Clinical Prediction Models

5 Efron B, Tibshirani RJ (1994) An Introduction to the Bootstrap. New York: Chapman & Hall

Supplemental Tables

Ordinal regression model (mRS 0-6 at 90 days)	adj. OR (95%CI)	P value
**ARWMC (periventricular)**		
Grade 1	1.28 (0.89 – 1.86)	0.176
Grade 2	1.17 (0.73 – 1.70)	0.607
Grade 3	1.56 (0.98 – 2.49)	*0.064*
**Cortical atrophy scale**		
Grade 1	1.38 (0.93 – 2.05)	0.106
Grade 2	1.75 (1.13 – 2.72)	**0.013**
Grade 3	3.19 (1.89 – 5.37)	**<0.001**
Age	1.01 (1.00 – 1.02)	0.046
Sex	0.93 (0.74 – 1.17)	0.526
Laterality (right vs. left)	1.02 (0.81 – 1.28)	0.860
Diabetes	1.03 (0.73 – 1.44)	0.871
CHD	1.35 (1.04 – 1.76)	0.025
**Hypertension**	0.64 (0.48 – 0.85)	**0.002**
Atrial Fibrillation	0.92 (0.73 – 1.15)	0.449
Dyslipidemia	0.89 (0.69 -1.13)	0.330
i.v. rtPA	0.89 (0.71 – 1.13)	0.349
**Baseline NIHSS**	1.10 (1.08 – 1.12)	**< 0.001**
**Pre-morbid mRS**	1.66 (1.51 – 1.84)	**< 0.001**
**Baseline ASPECTS**	0.80 (0.75 – 0.86)	**< 0.001**
Glucose	1.01 (1.00 – 1.01)	**< 0.001**
HbA1c	1.09 (0.95 – 1.23)	0.251
**Time from onset to first imaging**	1.00 (1.00 -1.00)	**0.005**

Supplemental Table S1. Ordinal logistic regression model for prediction of the ordinal mRS outcome at 90 days after EVT. ARWMC, Cortical atrophy, Hypertension, Baseline NIHSS, pre-morbid mRS, baseline ASPECTS and time from onset to first imaging exam were all shown to be independent predictors for clinical outcome, as listed above.

	Baseline model		Post-EVT		Post-discharge	
**Logistic regression**	**adj. OR (95%CI)**	**P value**	**adj. OR (95%CI)**	**P value**	**adj. OR (95%CI)**	**P value**
ARWMC basal ganglia						
Grade 1	1.18 (0.81,1.74)	0.576	1.17 (0.79,1.72)	*0.570*	1.11 (0.71,1.72)	*0.776*
Grade 2	1.25 (0.74,2.12)		1.26 (0.73,2.16)		1.03 (0.56,1.88)	
Grade 3	1.76 (0.78,3.97)		1.81 (0.79,4.14)		1.55 (0.62,3.88)	
**ARWMC periventricular**						
Grade 1	0.8 (0.51,1.26)	**0.048**	0.75 (0.48,1.2)	*0.083*	0.65 (0.38,1.1)	*0.077*
Grade 2	1.21 (0.7,2.11)		1.12 (0.64,1.96)		0.98 (0.52,1.86)	
Grade 3	0.68 (0.34,1.35)		0.67 (0.34,1.36)		0.59 (0.27,1.3)	
**Cortical atrophy scale**						
**Grade 1**	**0.73 (0.46,1.16)**	**0.002**	**0.73 (0.45,1.17)**	**0.002**	**0.57 (0.33,0.99)**	**< 0.001**
**Grade 2**	**0.54 (0.31,0.92)**		**0.52 (0.3,0.89)**		**0.36 (0.19,0.69)**	
**Grade 3**	**0.28 (0.14,0.57)**		**0.28 (0.14,0.57)**		**0.21 (0.09,0.48)**	
Age	1.00 (0.99,1.02)	0.589	1.01(0.99,1.02)	0.529	0.99 (0.98,1.02)	0.945
Sex	1.00 (0.74,1.35)	0.999	1.03 (0.76,1.40)	0.837	1.25 (0.89,1.77)	0.201
Laterality (right vs. left)	1.13 (0.84,1.51)	0.427	1.1 (0.82,1.49)	0.521	0.96 (0.69,1.35)	0.829
Diabetes	0.88 (0.55,1.4)	0.582	0.85 (0.53,1.37)	0.500	0.76 (0.44,1.31)	0.328
CHD	0.85 (0.6,1.21)	0.365	0.87 (0.61,1.25)	0.461	0.87 (0.58,1.3)	0.490
Hypertension	1.28 (0.89,1.85)	0.173	1.3 (0.9,1.88)	0.164	1.4 (0.92,2.13)	0.117
Atrial Fibrillation	1.3 (0.96,1.77)	0.088	1.26 (0.92,1.73)	0.143	1.1 (0.77,1.56)	0.610
Dyslipidemia	0.88 (0.63,1.22)	0.441	0.84 (0.6,1.17)	0.307	0.78 (0.54,1.12)	0.178
i.v. rtPA	1.34 (0.98,1.82)	0.064	1.35 (0.99,1.84)	0.061	1.4 (0.99,1.99)	0.057
Baseline NIHSS	0.99 (0.97,1.01)	0.393	1.00 (0.98,1.03)	0.705	0.99 (0.97,1.02)	0.717
**Pre-morbid mRS**	**0.7 (0.61,0.81)**	**< 0.001**	**0.7 (0.61,0.81)**	**< 0.001**	**0.48 (0.40,0.58)**	**< 0.001**
Baseline ASPECTS	1.08 (0.98,1.19)	0.112	1.02 (0.92,1.13)	0.718	1.1 (0.98,1.24)	*0.106*
Glucose	1.00 (0.99,1.01)	0.217	1.00 (0.99,1.01)	0.098	1.01 (1.00,1.01)	0.020
HbA1c	0.96 (0.79,1.16)	0.663	0.98 (0.81,1.2)	0.867	0.95 (0.77,1.17)	0.605
onset to first image (t)	0.99 (0.99,1.00)	0.586	0.99 (0.99,1.00)	0.790	0.99(0.99,1.00)	0.506
Recanalization			0.84 (0.48,1.47)	0.548	0.85 (0.45,1.63)	0.636
**TICI ≥ 2b**			**0.56 (0.4,0.78)**	**< 0.001**	**0.56 (0.39,0.81)**	**0.002**
NIHSS at 24 hours			0.99 (0.97,1.01)	0.157	0.98 (0.95,1.01)	0.230
Intracranial Bleeding			1.5 (0.78,2.9)	0.237	1.66 (0.78,3.53)	0.191
Puncture to TICI (t)			1.00 (0.99,1.00)	0.715	0.99 (0.99,1.00)	0.229
Number of manoeuvrers			0.99 (0.9,1.09)	0.828	0.98 (0.88,1.1)	0.742
**Follow-up ASPECTS**			**1.13 (1.04,1.23)**	**0.005**	1.1 (0.99,1.22)	*0.073*
**NIHSS at discharge**					**0.83 (0.8,0.86)**	**< 0.001**
**mRS at discharge**					**4.39 (3.4,5.68)**	**< 0.001**

Supplemental Table S2. Logistic regression models to predict mRS improvement after hospital discharge with either Baseline, Baseline + Post-EVT, und Baseline + Post EVT + Post Discharge metrics. Cortical atrophy is shown to be an independent predictor in all three models.

Supplemental Figures

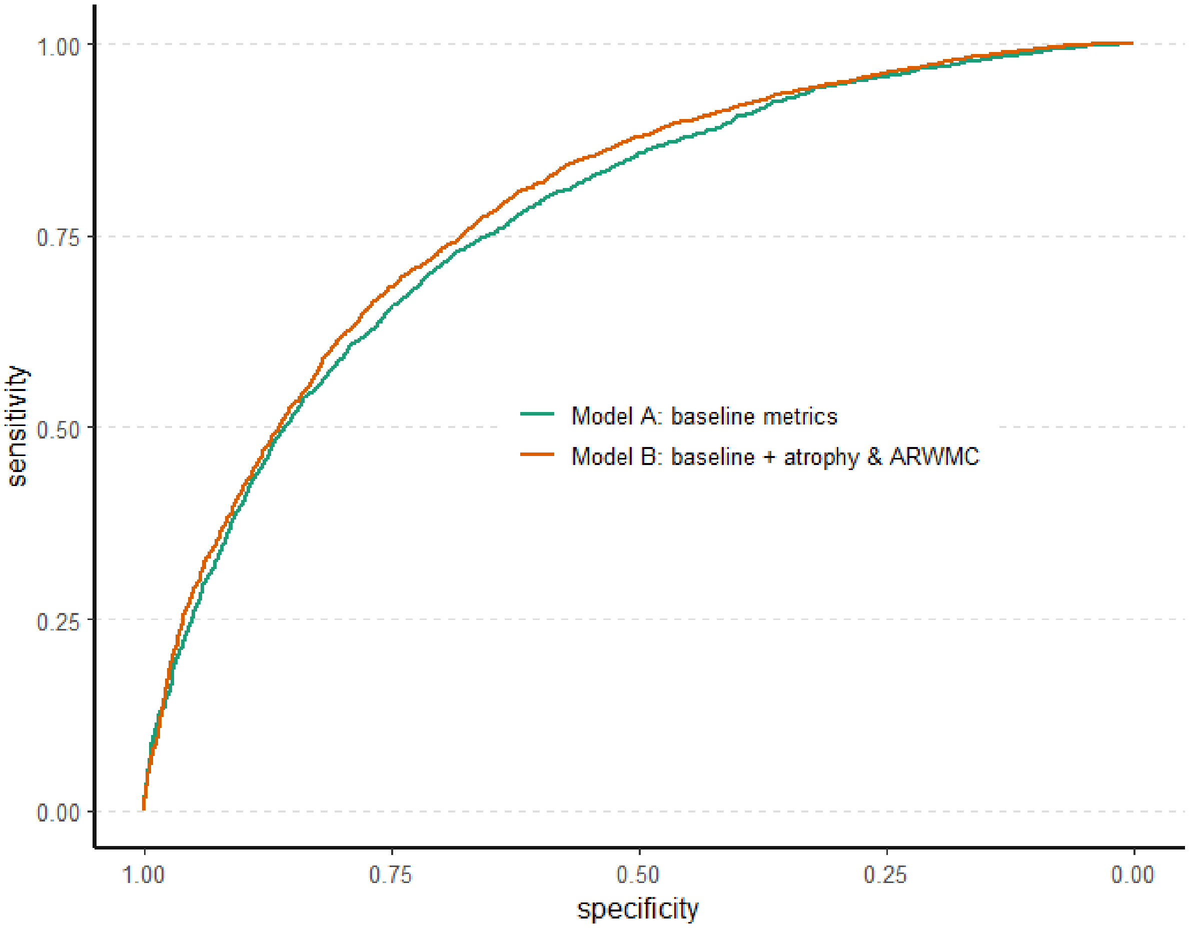

Supplemental Figure S1. ROC curves for the machine-learning classifiers constructed to predict favorable vs. unfavorable clinical outcome at 90 days through baseline metrics. The addition of cortical atrophy scale and ARWMC led to a minor improvement in the prediction performance (p<0.001).

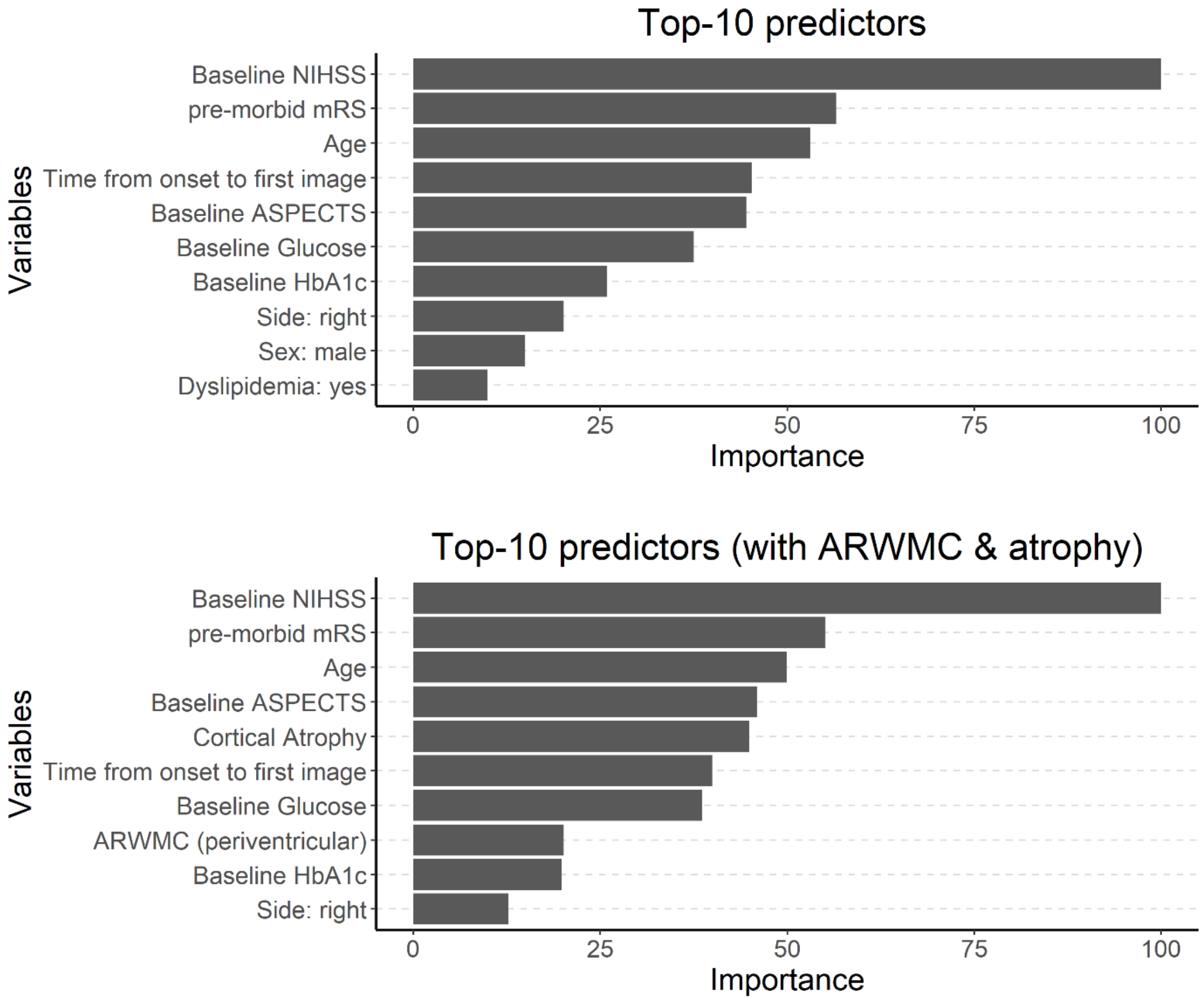

Supplemental Figure S2. Variable importance of predictors within the two developed machine learning classifiers without (top) and with (bottom) the inclusion of ARWMC and cortical atrophy; the two predictors after inclusion appear among the top predictors of clinical outcome.
